# Burdock-Derived Composites Based on Biogenic Gold, Silver Chloride and Zinc Oxide Particles as Green Multifunctional Platforms for Biomedical Applications and Environmental Protection

**DOI:** 10.3390/ma16031153

**Published:** 2023-01-29

**Authors:** Irina Zgura, Nicoleta Badea, Monica Enculescu, Valentin-Adrian Maraloiu, Camelia Ungureanu, Marcela-Elisabeta Barbinta-Patrascu

**Affiliations:** 1 National Institute of Materials Physics, Atomistilor 405A, 077125 Magurele, Romania; 2 General Chemistry Department, Faculty of Chemical Engineering and Biotechnologies, University “Politehnica” of Bucharest, 1-7, Polizu Street, 011061 Bucharest, Romania; 3 Department of Electricity, Solid-State Physics and Biophysics, Faculty of Physics, University of Bucharest, 405 Atomistilor Street, P.O. Box MG-11, 077125 Magurele, Romania

**Keywords:** “green” synthesis, burdock (*Arctium lappa* L.), biogenic metal and semiconducting nanoparticles, composites, antioxidant activity, antibacterial action, photocatalytic properties

## Abstract

Green nanotechnology is a rapidly growing field linked to using the principles of green chemistry to design novel nanomaterials with great potential in environmental and health protection. In this work, metal and semiconducting particles (AuNPs, AgClNPs, ZnO, AuZnO, AgClZnO, and AuAgClZnO) were phytosynthesized through a “green” bottom-up approach, using burdock (*Arctium lappa* L.) aqueous extract. The morphological (SEM/TEM), structural (XRD, SAED), compositional (EDS), optical (UV–Vis absorption and FTIR spectroscopy), photocatalytic, and bio-properties of the prepared composites were analyzed. The particle size was determined by SEM/TEM and by DLS measurements. The phytoparticles presented high and moderate physical stability, evaluated by zeta potential measurements. The investigation of photocatalytic activity of these composites, using Rhodamine B solutions’ degradation under solar light irradiation in the presence of prepared powders, showed different degradation efficiencies. Bioevaluation of the obtained composites revealed the antioxidant and antibacterial properties. The tricomponent system AuAgClZnO showed the best antioxidant activity for capturing ROS and ABTS•^+^ radicals, and the best biocidal action against *Escherichia coli*, *Staphylococcus aureus* and *Pseudomonas aeruginosa*. The “green” developed composites can be considered potential adjuvants in biomedical (antioxidant or biocidal agents) or environmental (as antimicrobial agents and catalysts for degradation of water pollutants) applications.

## 1. Introduction

Metal nanoparticles (MNPs) are widely used in engineering, and more in biomedical field (e.g., biosensors, diagnostic imaging, and drug delivery applications) [1,2]. They have attracted the interest of the scientific community due to their great potential in nanoscience [3].

MNPs (Ag [4], Cu [5], Au [6], Pt [7], Zn [8]) have received increasing attention in various fields such as electronics [8,9,10,11], optics [12] and biomedicine [6,13], due to their properties, e.g., the very large specific surfaces of MNPs that determine their catalytic properties [14]. This has led to the active use of MNPs as catalysts for a variety of industrial processes, e.g., environmental cleaning [15,16]. However, MNP use is difficult because they are complicated to handle and tend to agglomerate easily. This has the effect of reducing their specific surface area. Through aggregation, the excellent functionalities of MNPs are greatly diminished. That is why the researchers have attached catalytic MNPs on different support materials, such as polymeric materials [17,18] but also on metal oxide powders [6,18]. Nanomaterials based on metals (e.g., Ag, Cu, Au, Pt, Zn, Mg, etc.) and metal oxides (e.g., TiO_2_, Ag_2_O, ZnO, etc.) have also shown therapeutic benefits in the biomedical field [19,20,21,22]. In materials science, zinc oxide (ZnO) is one of the most used n-type semiconducting metal oxides, due to its tunable and multifunctional morphological and photonic properties [23]. Moreover, ZnO-based materials have been recognized to have low toxicity, photocatalytic activity, and antimicrobial properties [24]. The scientific literature presents various methods of obtaining these materials and various bi-/tricomponent composites, and the interest in composites arises due to the fact that when they are combined, they tend to demonstrate new or stronger characteristics, different from those presented by their individual components [25].

Most chemical methods are expensive and can produce potential risks for the environment and living organisms. Therefore, the interest in obtaining materials through green chemistry is increasing exponentially.

Valorization in a “green” manner, using the green chemistry principles, of the nature-derived raw materials and converting them into valuable materials is a good aspect to keep clean the environment. Plants have gained huge interest in nanotechnology since they are available, cost-effective, ecofriendly, and renewable. The vegetal bioconstituents (such as carbohydrates, polyphenolic compounds, flavonoids, steroids, sapogenins, tannins, terpenoids, polyols, proteins, etc.) [26] act as capping and stabilizing agents for the “green” synthesis of metal nanoparticles, and also give them interesting features such as antioxidant/antimicrobial/biocompatibility and also ecofriendliness.

Recent research in the field of green chemistry has evolved in the sense of obtaining bi-/trimetallic systems in the micro- and nanocomposite forms. DJ da Silva et al. demonstrated that the Ag/ZnONPs system exhibits antimicrobial properties against *Escherichia coli* and *Staphylococcus aureus*, antiviral activity against Delta SARS-CoV-2, and superior photocatalytic properties to AgNP and ZnONP nanoparticles [4]. The green system of ZnO/AgNPs presents anti-inflammatory activity and increases collagen fiber production when being successfully used to treat skin lesions [27]. Additionally, this system synthesized by using *Zingiber officinale* extract showed the highest cytotoxicity activity against human cancerous cell lines (breast, colon, and peripheral blood mononuclear cells) [28].

The bimetallic Au@ZnO nanorod system, based on the leaf extract of *Ziziphus jujuba*, has a 3–4 times higher photocatalytic activity in the photodegradation of industrial textile effluent and methyl orange compared to ZnO nanorods and to AuNPs [29].

Anjum et al. reported, for the first time, the synthesis of hybrid bicomponent nanoparticles (Au-AgNPs, AgNPs-ZnO, and AuNPs-AgNPs) using the same vegetal extract (*Manilkara zapota* leaf extract). This extract has biocompatibility with human cells, and presents antitumoral, antidiabetic, antibacterial, and antiglycation activities and enhanced therapeutic activity as compared to their monometallic components [30].

The green trimetallic nanocomposites based on Cu-Cr-Ni NPs [31] and CuO-Cr_2_O_3_-NiO [32] with antibacterial properties against *Staphylococcus aureus*, *Escherichia coli*, and *Bacillus cereus* showed superior antibacterial activity as compared to single-metal nanoparticles.

However, the present paper describes a new biogenic approach to synthesize burdock-derived metallic and semiconducting multicomponent particles (AuAgClZnO) with many interesting properties. The main objective of our work was the “green” design of AuAgClZnO composite particles, through a bottom-up approach, and their complex characterization. For the first time, the same extract (burdock extract) was used to obtain mono-, bi-, and trimetallic systems of Au, Ag, and ZnO. In order to achieve this aim, some specific objectives were followed:

(i) In the first step, biogenic monocomponent particles were prepared. For this purpose, the burdock aqueous extract was used as a precursor for “green” particles of gold, silver chloride, and zinc oxide (AuNPs, AgClNPs, and ZnO, respectively).
(ii) The developed monocomponent particles were further used as building blocks to achieve bi- (AuZnO and AgClZnO) and tricomponent (AuAgClZnO) particles.
(iii) The complex characterization of AuAgClZnO composite as compared to AuNPs, AgClNPs, ZnO, AuZnO, and AgClZnO.

In this study, we chose burdock (*Arctium lappa* L., Asteraceae family) because it is one of the most popular and widespread plants in our country (Romania) and is used in traditional medicine as a carminative, diuretic, depurative, antioxidant, anti-inflammatory, antibacterial, and antitubercular agent to treat many diseases (e.g., skin disorders, atherosclerosis, hepatitis, hypertension, and geriatric diseases). Its high therapeutic potential is determined by the presence of many polyphenolic compounds [33]. *Arctium lappa* leaves contain valuable bioactives such as phenolic acids (e.g., chlorogenic acid, caffeic acid, and cynarin), quercetin, quercitrin, arctiin, rutin, and luteolin [34].

In this work, the biosynthesized materials were physicochemically characterized, and their bioperformance (antioxidant and antibacterial properties) was further studied. Moreover, their catalytic activity for the degradation of Rhodamine B (RhB) as a model of a water pollutant was also assessed.

## 2. Materials and Methods

The present study focuses on the environmentally friendly synthesis of the metal and semiconducting particles (AuNPs, AgClNPs, ZnO, AuZnO, AgClZnO, and AuAgClZnO) by using burdock (*Arctium lappa* L.) aqueous extract and their complex characterization. The synthesis of the composites consists of two steps: (i) the preparation of AgClNPs and AuNPs, and (ii) the AgClNPs, AuNPs, or their mixture suspension further used to generate ZnO particles through the reaction between Zn(NO_3_)_2_ and NaOH.

All the chemical substances—AgNO_3_, HAuCl_4_·3H_2_O, Zn(NO_3_)_2_·6H_2_O, NaOH Tris[hydroxymethyl] aminomethane, sodium chloride, 2,2-azinobis-(3-ethyl benzthizoline-6-sulfonic acid) (ABTS), HCl, Luminol (5-Amino-2,3-dihydro-1,4-phthalazinedione), H_2_O_2_, K_2_S_2_O_8,_ Trolox (6-hydroxy-2,5,7,8-tetramethyl chroman-2-carboxylic acid) and Rhodamine B—were purchased from Sigma Aldrich (Munich, Germany) and were used without any further purification.

### 2.1. Preparation of AgClNPs and AuNPs by Using the Burdock Extract

The aqueous burdock extract (EB) was made from fresh leaves of *Arctium lappa* which were inserted into hot distilled water (in a mass ratio *vegetal material*: *water* = 1:5, *w*/*w*) as previously described [33].

The silver and gold nanoparticles were prepared in two glass beakers, each containing 50 mL of EB. In the first beaker, AgNO_3_ was introduced, and in the second, HAuCl_4_∙3H_2_O, under continuous stirring in the dark for 24 h (VIBRAX stirrer, Milian, OH, USA, 200 rpm), until the final concentrations reached the values of 2.6 mM (for AgNO_3_) and 0.8 mM (for HAuCl_4_). After 45 min., the color of the mixtures turned from pale yellowish to brown (1st beaker) and to purple (2nd beaker), highlighting the formation of silver chloride nanoparticles (sample AgClNPs) and gold nanoparticles (sample AuNPs), respectively. The phytocompounds present in burdock extract helped to form the nanoparticles by giving up electrons. The vegetal extract acted both as a bioreducing and capping agent for NP formation.

### 2.2. Preparation of Composites Based on ZnO, AgClNPs, AuNPs, and a Mixture of AuNPs-AgClNPs in Burdock Extract

Aqueous solutions of 0.5 M of Zn(NO_3_)_2_ and of 1 M of NaOH were dropwise added (under continuous stirring for 30 min) to burdock extract (EB) or to suspensions of AuNPs, AgClNPs, and a mixture of AuNPs/AgClNPs. In each case, the resulting precipitates were centrifuged and washed several times, and then dried in vacuum at 100 °C for 2 h. The obtained nanostructures were named as follows: ZnO, AuZnO, AgClZnO, and AuAgClZnO, respectively.

The preparation of the burdock-derived composites involved the following steps:

(i) HAuCl_4,aq_ + EB (phyto-compounds) → AuNPs;
(ii) AgNO_3,aq_ + EB (phyto-compounds) → AgClNPs;
(iii) Zn(NO_3_)_2_ + 2NaOH → Zn(OH)_2_↓ + 2NaNO_3_;
(iv) EB/AuNPs/AgClNPs/(AuNPs + AgClNPs) + Zn(OH)_2_$\overset{{100\;}^{{}^{\circ}}C}{\to }\;$ZnO/AuZnO/AgClZnO/AuAgClZnO + H_2_O.

The two-step “green” synthesis of phyto-ZnONPs involves:

(i) Zn(NO_3_)_2_ + 2NaOH → Zn(OH)_2_↓ + 2NaNO_3_;
(ii) EB + Zn(OH)_2_$\overset{{100\;}^{{}^{\circ}}C}{\to }$$\mathrm{ZnO}\;(\mathrm{capped}\;\mathrm{with}\;\mathrm{phyto}\text{-}\mathrm{molecules})+H_{2}O$.

The synthesized materials were evaluated from the point of view of morphological, physical stability, compositional, structural, optical, photocatalytical, and biological (antioxidant and antibacterial) properties.

### 2.3. Physicochemical and Biological Characterization of “Green” Developed Composites

The UV-Vis absorption spectra of the obtained materials were recorded on a double-beam UV–Vis 670 Jasco spectrophotometer (Jasco, Tokyo, Japan), in the 200–800 nm wavelength range, operated at a resolution of 1 nm. Fourier-transform infrared (FTIR) spectra were recorded in the range of 4000–500 cm^−1^, with a resolution of 4 cm^−1^ in transmittance mode, on Perkin Elmer-Spectrum 100. The morphological properties of composite powders were evaluated using a Carl Zeiss (Oberkochen, Germany) Gemini 500 field emission scanning electron microscope (FESEM) working in high vacuum (HV), from 0.2 to 30 kV, and equipped with a LaB6 emitter, InLens, and SE2 detectors. The elemental composition of the samples was evaluated using a Bruker QUANTAX 200 energy-dispersive X-ray spectrometer detector (EDS) with Peltier cooling and an energy resolution < 129 eV at Mn-Ka.

The crystalline phase of the powders was identified with X-ray diffraction (XRD) with a Bruker D8 Advance diffractometer (Bruker AXS, Karlsruhe, Germany) with CuKα radiation (λ = 0.154 nm); using a nickel filter, the Kβ radiation was removed. Diffraction patterns were recorded in Bragg–Brentano geometry in the 2θ range from 20° to 80° at a rate of 0.6°/min (2θ/min). The obtained XRD data were processed using “Bruker Diffrac plus Basic Package Evaluation v.12”.

Morphostructural investigations were carried out using an atomic resolution analytical JEM ARM200F (JEOL Ltd., Tokyo, Japan) microscope operated at 200 kV acceleration voltage and equipped with a JEOL JED-2300T unit for energy-dispersive X-ray spectroscopy (EDS) analysis. The samples in powder form were gently crushed in an agate mortar and dispersed in ethanol. A droplet of the suspension was then deposited onto a 400-mesh carbon lacey TEM Cu grid and allowed to dry at room temperature.

The photocatalytic properties of the composites were determined by evaluating the photodegradation of Rhodamine B (RhB) aqueous solutions during the irradiation with solar light. Approximately 0.5 mg of composite powders were immersed in a beaker containing 10 mL of RhB aqueous solution, with a concentration of 2 × 10^−5^ M and prepared using deionized water (Millipore system). The beaker was irradiated with solar light obtained using an SF300-A Small Collimated Beam Solar Simulator (Sciencetech, London, ON, Canada) equipped with an Air Mass AM1.5G Filter (spot size: 25 mm diameter at one Sun) and an integrated electrical shutter with a controller and a Xe lamp (300 W). At different time intervals, approximately 3 mL of dye solutions was carefully extracted from the beaker and placed in a cuvette, and the optical absorbance spectra in the spectral domain 400–700 nm were measured (UV–Vis–NIR CARY 5000 spectrophotometer, Varian, Agilent Technologies Deutschland GmbH, Waldbronn, Germany). The degradation of Rhodamine B was evaluated by monitoring the characteristic band peaking at ~554 nm.

By measuring the variation in the absorption peak at the wavelength of 554 nm, the degradation of RhB was determined. The dye degradation efficiency was estimated using the equation:

Degradation efficiency = (C_0_ − C)/C_0_ × 100 (1)

where C_0_ is the initial value of the dye concentration; C is the value of the dye concentration at time t.

### 2.4. Evaluation of Particle Size Distribution

The particle size (Zav) and polydispersity index (PdI) were analyzed with dynamic light scattering (DLS) using a Zetasizer Nano ZS, Malvern Instruments Inc., Worcestershire, UK. The particles size was calculated using the Stokes–Einstein equation (Equation (2)). All the measurements were performed in triplicate and the results were expressed as mean ± S.D.

(2) $$d=\frac{kT}{3\pi \rho D}$$

where d = the diameter of the spherical particle, k = Boltzmann constant, T = temperature, ρ = the viscosity of the medium, and D = the diffusion coefficient of the particle.

### 2.5. Electrokinetic Potential Analysis

The physical stability of the samples was evaluated by measuring the electrophoretic mobility in an electric field using the Zetasizer Nano ZS, Malvern Instruments Inc., Worcestershire, UK. The zeta potential (*ξ*) was calculated using the Helmholtz–Smoluchowski Equation (3):

(3) $$\xi =EM\cdot \frac{4\pi \eta }{\varepsilon }$$

where *ξ* is the zeta potential, *η* viscosity of the dispersion medium, *EM* is electrophoretic mobility, and *ε* is the dielectric constant. The conductivity of samples was adjusted using 50 μL of NaCl 0.9% solution and all the measurements were performed in triplicate.

### 2.6. Biological Characterization of Developed Materials

#### 2.6.1. In Vitro Antioxidant Activity Analysis

The radical scavenger activity of the nanoparticle samples and the burdock extract was evaluated using the ABTS and the chemiluminescence methods. The cation radicals, ABTS^●+^, were generated from the reaction between 2,2-azinobis-(3-ethyl benzthizoline-6-sulfonic acid) solution (7 mM) and K_2_S_2_O_8_ solution (2.45 mM) after 16 h in dark conditions. The work solution was normalized to the absorbance 0.70 ± 0.01 at λ = 734 nm. The reference solution was prepared using 3 mL of ABTS^●+^ work solution and 2 mL of ultra-purified water, and the sample was prepared identically, by replacing 1 mL of ultra-purified water with the 1 mL of nanoparticle suspension. The samples were analyzed at 4 min after mixing at 734 nm using UV-Vis Spectrophotometer Type V670, (Jasco, Tokyo, Japan). The inhibition capacity (%) was calculated using Equation (4):

(4) $$\%Inhibition{\;\mathrm{ABTS}}^{•+}=\frac{A_{0}-A_{S}}{A_{0}}\times 100$$

where A_0_ = the absorbance of the blank (unscavenged radical cation solution); As = the absorbance of the nanoparticle suspension.

The chemiluminescence system used to evaluate of the scavenger activity for the short-life radicals was formed by 0.01 M of luminol solution, H_2_O_2_ (10^−5^ M), and Tris–HCl buffer solution (pH = 8.6). The free-radical-scavenger activity of the samples was calculated by applying the relation (5) and using a Turner Design TD 20/20 chemiluminometer (Sunnyvale, CA, USA)

(5) $$\mathrm{AA}\;(\%)=\frac{I_{0}-I_{S}}{I_{0}}\times 100$$

I_0_ = the maximum CL for reference at t = 5 s; Is = the maximum CL for sample at t = 5 s [35].

#### 2.6.2. In Vitro Antibacterial Activity Analysis

*Minimum Inhibitory Concentration (MIC) determination*

To evaluate the antibacterial activity, the AuNPs, AgClNPs, ZnO, AuZnO, AgClZnO, and AuAgClZnO were tested against three pathogenic microbial strains: *Escherichia coli* ATCC 8738, *Pseudomonas aeruginosa* ATCC 15442 (Gram-negative bacteria) and *Staphylococcus aureus* ATCC BAA 1026 (Gram-positive bacteria).

Minimum inhibitory concentration represents the lowest concentration of an antimicrobial agent at which the growth of microorganisms is totally inhibited, expressed in µg/mL. For MIC determination, EUCAST [36] mostly recommends broth microdilution, except for fosfomycin and mecillinam, for which it recommends agar dilution. To determine MIC values, all quantitative methods use Mueller–Hinton broth (MHB) [37,38]. All reagents used for antimicrobial investigations were purchased from VWR (Darmstadt, Germany).

Serially diluted logarithmic concentrations of prepared burdock-derived samples ranging from 400 to 0.195 μg/mL were inoculated with standardized overnight cultures of the cultured bacteria at 37 °C.

MIC values for each microorganism were reported as the median of three experiments. Standard deviation (S.D.) was calculated as the square root of variance using STDEV function in Excel 2010.

The antibacterial activity of the burdock-derived samples was determined employing the Kirby–Bauer test. The method is based on the property of the antimicrobial agent to diffuse into a solid culture medium on which the bacterial culture to be tested is seeded. Briefly, the melted and cooled culture medium (Luria Bertani Agar acc. Miller medium) at 50 °C was poured into 4 mm thick Petri dishes, and then it was used after solidification. A bacterial suspension with a concentration close to 0.5 on the MacFarland scale was made. After this step, wells were made with a sterile glass tube into which 50 µL of each tested sample was pipetted. Then, agar Petri dishes were incubated under suitable conditions at 37 °C for 18–24 h. The diameter of the zone of inhibition (ZOI, mm) was measured [39,40].

## 3. Results and Discussion

### 3.1. Optical Characterization of Phytoderived Materials

**Figure 1:**
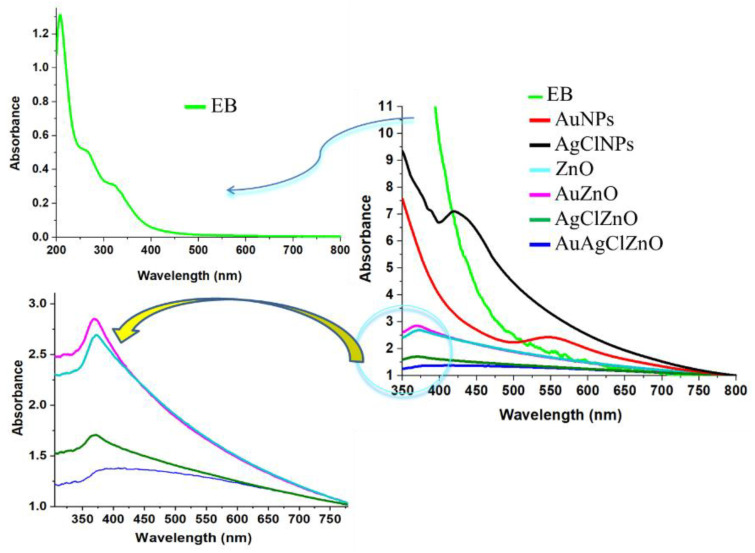
Comparative presentation of UV-Vis absorption spectra of burdock-derived samples. All spectra were normalized at 800 nm. The bottom inset shows the spectral fingerprint of ZnO particles. The top inset shows the spectrum of burdock extract (EB).

The formation of burdock-derived materials was observed by means of UV–Vis absorption spectroscopy as shown in Figure 1. ZnO-containing particles presented an absorption peak located at the wavelengths: 372, 369, and 371 nm for the samples of ZnO, AuZnO, and AgClZnO, respectively. This peak is characteristic for ZnO nanoparticles as mentioned by various reports [25,41,42,43,44], highlighting that ZnO generally shows UV absorption bands at the λmax ranging from 355 to 380 nm. The bottom inset of Figure 1 displays the spectral signature of ZnO in the spectra of the ZnO-based particles.

As observed, the AuNP spectrum presents a single absorption band at 547 nm, characteristic for spherical gold nanoparticles [45], while in the spectrum of AgClNPs, a band at 420 nm was observed, indicating the formation of AgClNPs [46]. Similar UV-Vis spectra were observed by Fageria et al. [47] for ZnO, ZnO/Au, and ZnO/Ag nanoparticles, where a ZnO peak at 369 nm was identified in these spectra.

UV-Vis absorption spectrum of burdock extract (see the top inset of Figure 1) shows the following peaks: at 208 nm attributed to the phenolic compounds and carbohydrates, and at 319 nm assigned to the B ring portion (cinnamoyl system, band I) of flavonoids. A shoulder at 263 nm was also observed, attributed to the absorption of amino acid residues of proteins and of the A-ring of flavonoids (benzoyl system, band II) [46,48].

The UV-Vis absorption spectrum of AuAgClZnO displays a very large/wide band between 350–750 nm with a peak centered at 411 nm, covering the absorption of all components of this composite.

**Figure 2:**
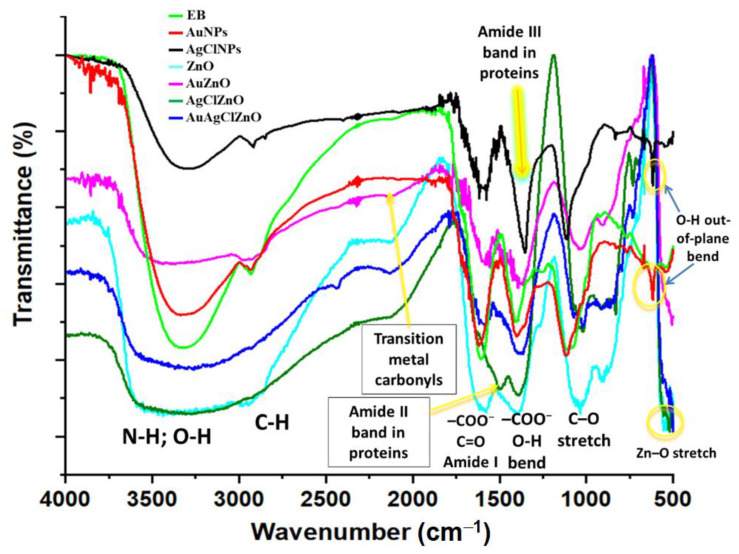
Comparative presentation of FTIR spectra of burdock extract and of burdock-derived particles.

The burdock-derived samples were further investigated with FTIR spectroscopy (Figure 2). The attributions of the main FTIR bands are shown in Table S1 (Supplementary Material) . An intense sharp band centered at 3321 cm^−1^, 3325 cm^−1^, and 3297 cm^−1^ observed in the spectrum of EB, AuNPs, and AgClNPs, respectively, is attributed to the bending and stretching vibrations of hydroxyl groups intermolecularly hydrogen bonded in alcohols, polysaccharides, and phenolic compounds/polyphenols; these bands are assigned also to the stretching vibrations of the primary and secondary amines [46]. This band widened in the case of ZnO-containing samples (ZnO, AuZnO, AgClZnO, and AuAgClZnO), overlapping the frequencies of the following groups: phenols, OH stretch, and hydrogen-bonded O–H (the bending and stretching vibrations of hydroxyl groups in alcohols, polysaccharides, and phenolic compounds) [46,49]. The samples EB, AuNPs, and AgClNPs presented FTIR bands originating from C–H anti-symmetric stretching vibration, in the wavenumber range of 2933–2923 cm^−1^, and also C–H symmetrical stretch vibration of alkyl chains, in the range of 2871–2848 cm^−1^ (see Table S1 in the Supplementary Material).

The peaks observed at 545 cm^−1^, 694 cm^−1^, 555/519 cm^−1^, and 559 cm^−1^ in the FTIR spectra of the samples of ZnO, AuZnO, AgClZnO and AuAgClZnO, respectively, originated from the Zn-O stretching vibration of the hexagonal phase of ZnO [42,50].

Other FTIR bands which are presented in the obtained burdock-derived samples were due to the carbonyl stretch and carboxylate groups in proteins, C-O bending in esters, –C–O–C– ether, phenol or tertiary alcohol, and OH bend (see Table S1 in the Supplementary Material).

As a consequence, the results of the FTIR analysis indicate that the functional groups belonging to the phytocompounds of burdock extract (proteins, carboxylates, flavones, alcohols, polyphenols, and ethers) may also act as reducing and capping agents for AuNPs, AgClNPs, and ZnO particles’ formation. These functional groups are present also on the surface of the particles AuZnO, AgClZnO, and AuAgClZnO. The presence of these hydroxyls, carboxylates, carbonyls, and other functional groups prevents agglomeration of the “green” prepared NPs in the aqueous extract medium as mentioned also by Rahman et al. [42].

The involvement of carboxylic acids, flavonoids, alcohols, and polyphenols in the bioreduction as well as the capping of bimetallic Ag-ZnO and Au-ZnO particles was also demonstrated via FTIR analysis by Anjum et al. [30] who used *Manilkara zapota* leaf extract as a precursor. This fact underlines the role of phytochemicals in the donation of electrons or hydrogen atoms for the bioreduction of metals, and in the stabilization of resulting metallic nanoparticles, granting them at the same time a high biocompatibility and superior biological activity [30].

### 3.2. Evaluation of Zeta Potential of the Phytometallic Particles

Zeta potential is an important parameter that evaluates the stability of nanoparticle systems, with the increasing zeta potential corresponding to an increase in the nanoparticles physical stability. In our case, the samples of AgClNPs, ZnO, and the mixture sample presented a very good stability having a zeta potential value less than −25 mV. These results are similar to those reported in the literature, when the silver nanoparticles based on marigold flower presented a physical stability of −27.1 mV [51] and −32.7 mV using rhamnogalacturonan gum [52]. The samples containing AuNPs showed a moderate stability, having a zeta potential of −18.5 mV for the individual sample and −17.6 mV for AuZnO, but a higher stability than the gold nanoparticles obtained using the aqueous extract of *Mangifera indica* seeds that presented a zeta potential value of −1.98 mV [53]. The three-component system AuAgClZnO showed good stability, having a value of the zeta potential equal to −29.8 ± 0.57 mV.

**Figure 3:**
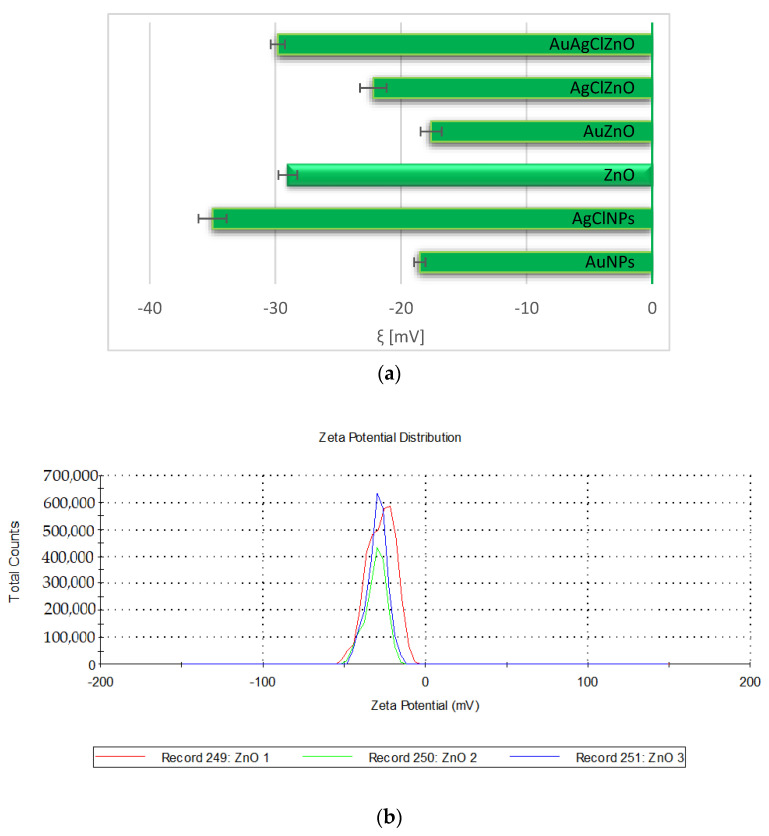
(**a**) Comparative presentation of the electrokinetic potential of samples; (**b**) The electrokinetic potential of ZnO sample.

Figure 3a displays a comparative presentation of the zeta potential values for the burdock-derived samples, and Figure 3b shows the distribution of zeta potential for the ZnO sample.

### 3.3. Structural Characterization

**Figure 4:**
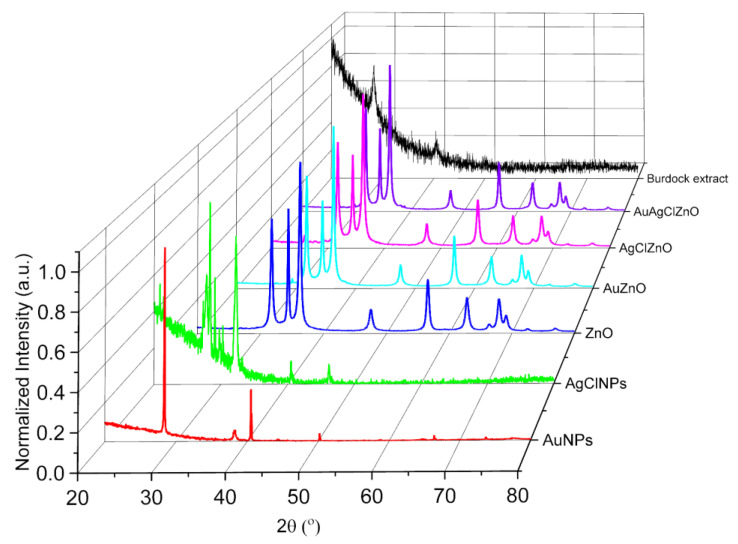
XRD patterns of the samples.

The crystal structure was established by the diffraction peaks shown in Figure 4. For the monocomponent samples, it was found: (i) for Au, the diffraction peaks positioned at 38.2° and 44.4° correspond to the Miller indices of the reflecting planes (111) and (200) assigned to the cubic phase (file 00-066-0091); (ii) the peaks at 27.8°, 32.2°, and 46.2° correspond to the crystallographic planes (111), (200), and (220) specific to the cubic unit cell of the AgCl crystal (file 00-031-1238), and (iii) in the case of ZnO, the diffraction peaks from 31.8°, 34.7°, 36.3°, 47.6°, 56.6°, 62.9°, 66.4°, 67.9°, and 69.1° correspond to the crystallographic planes (100), (002), (101), (102), (110), (103), (200), (112), and (201) and are attributed to the hexagonal phase of ZnO (file 00-036-1451).

In Figure 4, the diffractogram obtained from the burdock extract was also inserted. It can be seen that there are several diffraction maxima in this case as well; they were found at 28.3° and 40.5°, respectively, and correspond to the Miller indices of the reflective planes (200) and (220), respectively, of the cubic phase of KCl. These diffraction peaks were also found in the diffractograms obtained on the AuNP and AgClNP samples; these samples were kept as they are obtained in the burdock extract. For X-ray diffraction, these samples were deposited by drop casting on a Si wafer with no X-ray signal. In the XRD spectra of ZnO, AuZnO, AgClZnO, and AuAgClZnO powders, the diffraction peaks corresponding to the burdock extract were not found because the synthesis route of them involves the addition of two aqueous solutions to a volume of burdock extract with/without Au/AgClNPs, and in this way, the burdock extract is more diluted. In addition, the powders were washed several times, so the peaks related to KCl were not evident. The bi-/tricomponent samples showed mixed diffraction peaks from ZnO particles and Au/AgClNPs. Moreover, minor peaks of Au and AgCl and major peaks of ZnO were observed in the XRD patterns for bi- and trimetallic particles. This behavior in the XRD pattern was also observed for Ag–ZnO NPs and Au–ZnONPs obtained from *Manilkara zapota* leaf extract [30], as well as for the Ag–ZnO NPs prepared from *Silybum marianum* [54].

### 3.4. Size and Morphological Characterization of Phytmaterials

**Figure 5:**
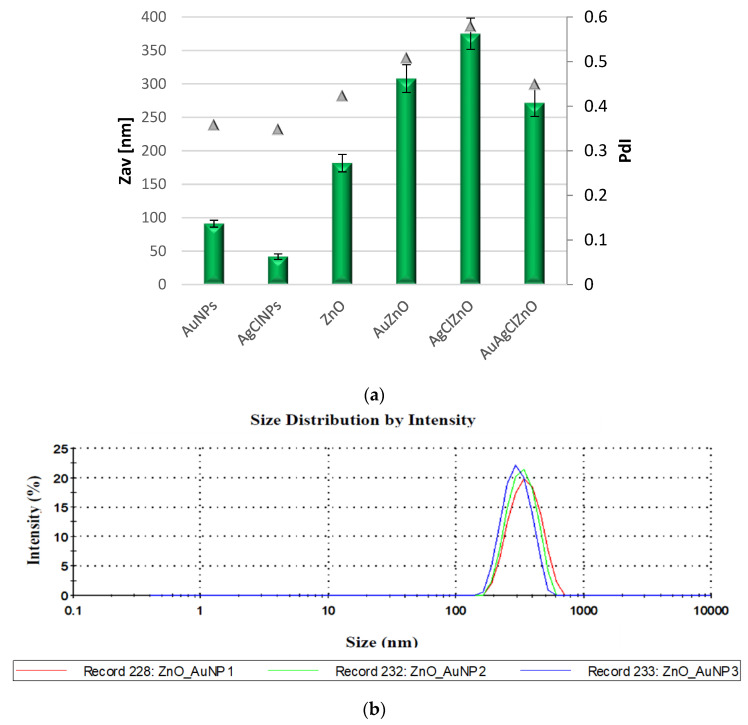
(**a**) The average particle size and PdI index of samples; (**b**) Size distribution of particles population for AuZnO sample.

The average particle size (Zav) and PdI index of samples were determined using light scattering measurements (Figure 5). The individual systems presented a size between 91–182 nm, and for the complex systems, showed a size between 272 and 375 nm with PdI index between 0.448–0.578 (Figure 5a). The average particle size of AuNPs, AgClNPs and ZnO was 91 nm, 41 nm and 181 nm, respectively. The sample AgClZnO showed a PdI of 0.57, indicating the existence of several populations of particles; this could also be due to the agglomeration phenomenon due to the potential value of −22.2 ± 1.06 mV. Additionally, the AuAgClZnO particles presented a mean dimension of 271 nm, having a large size as compared to individual systems. Figure 5b displays the size distribution of particle population for the AuZnO sample. The results obtained using DLS analyses were in concordance with the data obtained from SEM and TEM microscopy.

### 3.5. Morphological and Compositional Characterization

**Figure 6:**
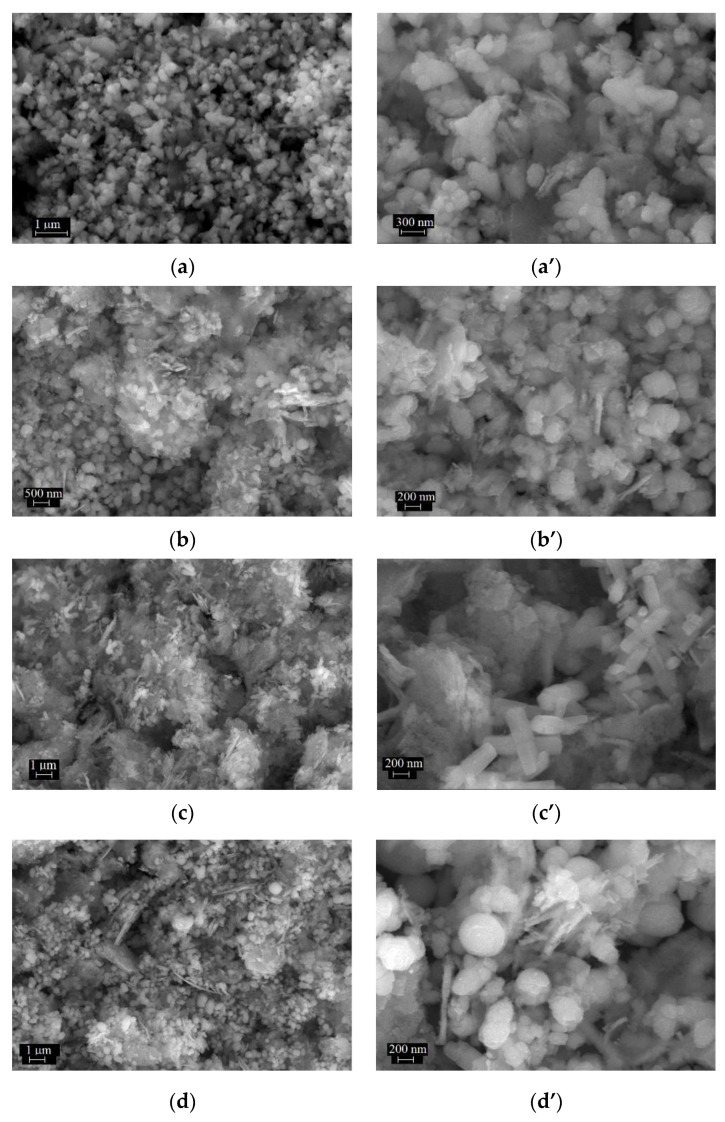
SEM images of samples at two magnifications, (**a**,**a’**) ZnO; (**b**,**b’**) AuZnO; (**c**,**c’**) AgClZnO; (**d**,**d’**) AuAgClZnO.

**Figure 7:**
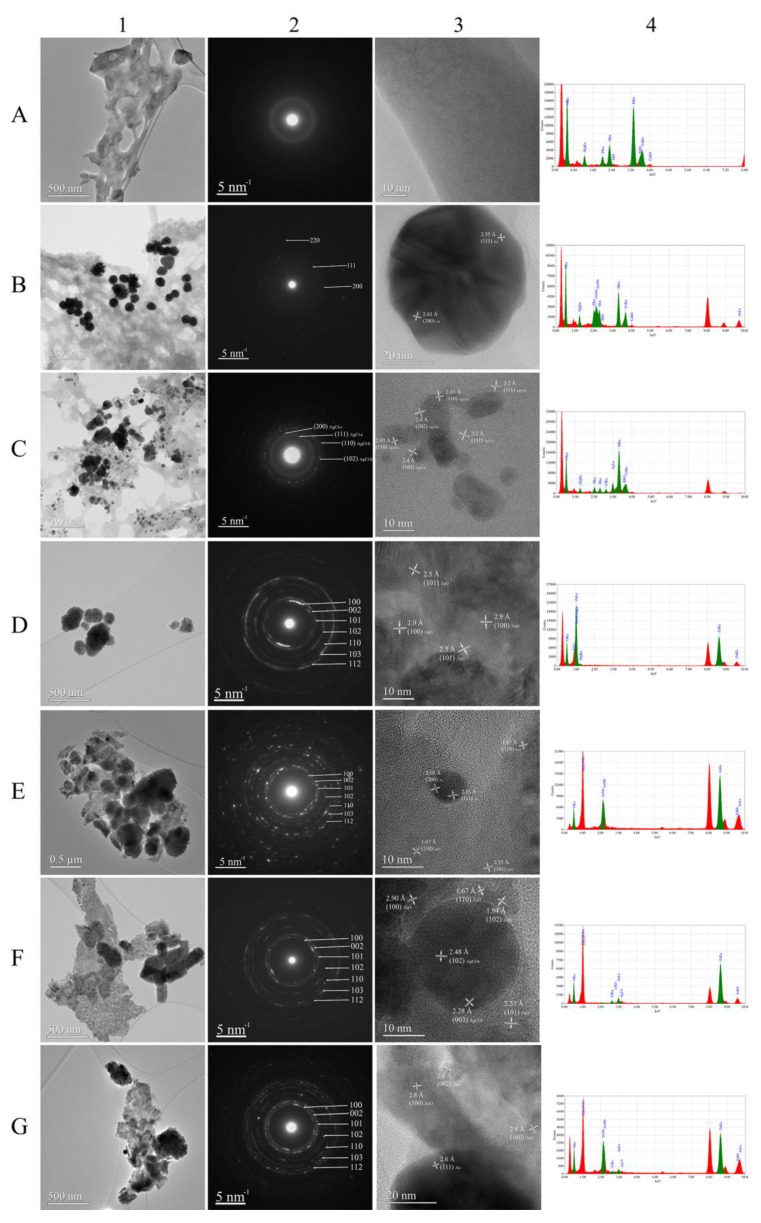
TEM results obtained using CTEM (**1**), SAED (**2**), HRTEM (**3**) and EDS (**4**) of samples for: burdock extract (**A**), AuNPs (**B**), AgClNPs (**C**), ZnO (**D**), AuZnO (**E**), AgClZnO (**F**) and AuAgClZnO (**G**).

The features of the samples were investigated from a morphological point of view by using the SEM and TEM techniques. Conventional TEM (CTEM), selected area electron diffraction (SAED) and high-resolution TEM (HRTEM) were used for morphologic and structural characterization while energy-dispersive X-ray spectroscopy (EDS) determined the chemical composition. Morphological analysis of ZnO powder showed the presence of a mixture of flower- and spindle-type structures, with dimensions ranging from 150 nm to 750 nm (Figure 6(a,a’) and Figure 7(D1,D3)). SEM and TEM analyses of AuNPs and AgClNPs samples revealed that the particles had a size between 6 nm and 89 nm for Au nanoparticles (Figure 7(B1)) and between 3 nm and 117 nm for AgClNPs (Figure 7(C1)), respectively. The composite samples had different morphologies as follows: (i) the AuZnO powder consisted of cylindrical structures with dimensions from 200 nm to 300 nm (Figure 6(b,b’) and Figure 7(E1)); (ii) the AgClZnO powder consisted of rod-type structures with a diameter of approximately 200 nm (Figure 6(c,c’) and Figure 7(F1)), and (iii) the three-component AuAgClZnO powder consisted of spherical and spindle-type structures with dimensions between 200 nm and 400 nm (Figure 6(d,d’) and Figure 7(G1)).

For the burdock sample (CTEM image, Figure 7(A1)), the SAED pattern (Figure 7(A2)) and the HRTEM image (Figure 7(A3)) show that it was all amorphous. The EDS spectrum (Figure 7(A4)) evidenced the presence of several chemical elements (O, Mg, P, S, K and Ca) beside Cu and C from the TEM grid.

In the case of the AuNP sample, the SAED pattern (Figure 7(B2)) confirmed that they were crystallized in a cubic structure. In the HRTEM image (Figure 7(B3)), lattice fringes of 2.35 Å and 2.03 Å characteristic of (111) and (200) cubic Au were clearly visible. The EDS spectrum (Figure 7(B4)) confirmed once more that Au was present in the sample, but it also showed the chemical elements present in the burdock sample.

For AgClNPs, the SAED pattern (Figure 7(C2)) and HRTEM image (Figure 7(C3)) demonstrate that the NPs were mostly crystallized in a cubic structure, but they also reveal the presence of the hexagonal phase of AgCl. This phase was not identified with XRD. The reason why this happened is that information obtained with TEM comes from a small area of a TEM grid containing an infinitesimal volume of sample compared with the one analyzed in XRD. This means that the signal given by hexagonal-phase AgCl can be lost in the background if the number of NPs with this crystalline structure is very small. The EDS spectrum (Figure 7(C4)) confirms the presence of Ag and Cl beside the chemical elements from the burdock extract.

The CTEM image of ZnO sample (Figure 7(D1)) show that NPs were agglomerated. The diffraction rings observed in the SAED pattern (Figure 7(D2)) correspond to hexagonal-phase ZnO. Lattice fringes of 2.9 Å and 2.5 Å characteristic of the (101) and (101) planes of hexagonal ZnO are visible in the HRTEM image (Figure 7(D3)). Only the peaks of Zn and O were present in the EDS spectrum (Figure 7(D4)). The hexagonal phase of ZnO particles was also observed in the FTIR spectra (see Section 3.1) and in XRD patterns (see Section 3.3), these results being similar to those for “green” synthesized ZnO nanoparticles using *Lactobacillus plantarum* [55].

For the last three samples (AuZnO, AgClZnO and AuAgClZnO), the SAED patterns (Figure 7(E2,F2,G2)) confirmed the presence of ZnO in a hexagonal structure. The absence of the characteristic diffraction spots for AuNPs and AgClNPs from the SAED patterns is explained by the fact that they were present in a very small amount in these samples. HRTEM was able to demonstrate that AuNPs (Figure 7(E3,G3)) and AgClNPs (Figure 7(F3)) were present in these samples. Additionally, the EDS spectra show characteristic peaks for Au (Figure 7(E4,G4)), Ag and Cl (Figure 7(F4,G4)) similar to those from the literature [56].

### 3.6. The Photocatalytic Properties of Obtained ZnO-Based Materials

**Figure 8:**
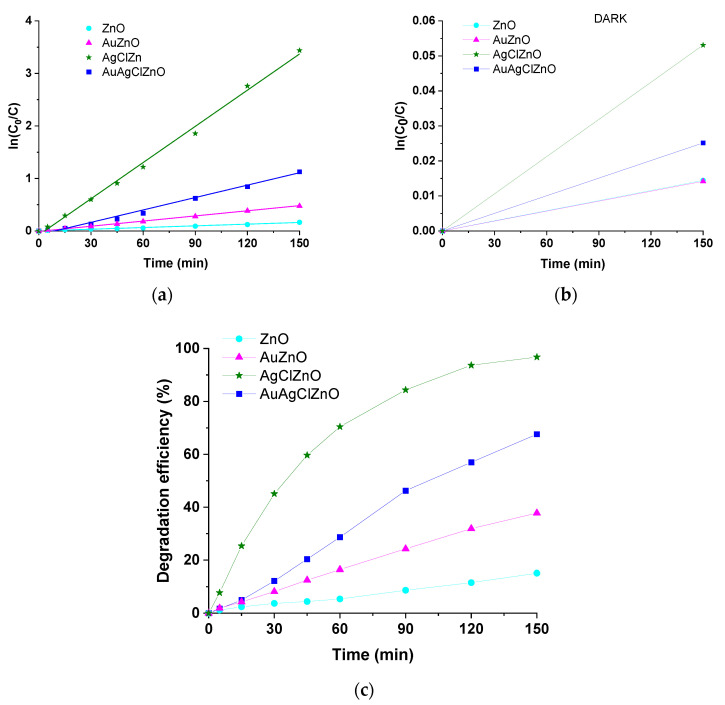
(**a**) ln(C_0_/C) vs. time for RhB degradation in the presence of the ZnO-based powders. The straight line represents the linear fit of the data; (**b**) ln(C_0_/C) vs. time for RhB degradation in the presence of the ZnO-based powders in dark conditions; (**c**) Time dependence of RhB degradation efficiency in the presence of ZnO-based powders.

The photocatalytic performance of the prepared powders of ZnO, AuZnO, AgClZnO and AuAgClZnO was examined for the degradation of Rhodamine B (RhB) under solar light irradiation. The RhB solution presented an absorption peak at 554 nm, and its intensity decreased under solar light irradiation in the presence of the synthesized ZnO-powders (Figure 8). Thus, in the presence of ZnO or AuZnO powders, the absorption peak at 554 nm of the RhB solution decreased slower when compared with the decrease observed in the presence of AgClZnO powder. Additionally, the presence of the three-component powder, AuAgClZnO, induced a more efficient degradation of RhB than the ones observed in the presence of the ZnO and AuZnO powder but much lower than in the case of AgClZnO. In the presence of AgClZnO, AuAgClZnO, AuZnO, and ZnO powders, the degradation percentages of RhB were 97.02%, 67.95%, 38.1% and 15.56%, respectively, after 150 min of solar light irradiation. These results demonstrate that the AgClZnO composite possessed much higher photocatalytic efficiency compared to the ZnO sample. It should be mentioned that all the composites had a better photocatalytic activity than ZnO particles, and also, as can be seen from Figure 8b, the photodegradation of RhB in the presence of the tested powders but in the absence of light was lower (~5% for the AgClZnO powder and less than 3% for the rest of the samples). By fitting the experimental results with a function of the first degree (Figure 8a), it proves that the mechanism is of the pseudo-first-order, according to the kinetics data. By fitting the experimental curves with a linear regression curve (pseudo-first-order, ln(C_0_/C) = kt) (Figure 8a), the reaction rate constant (k) was calculated, and the obtained values given in Table 1 being in good agreement with the literature [56,57].

**Figure 9:**
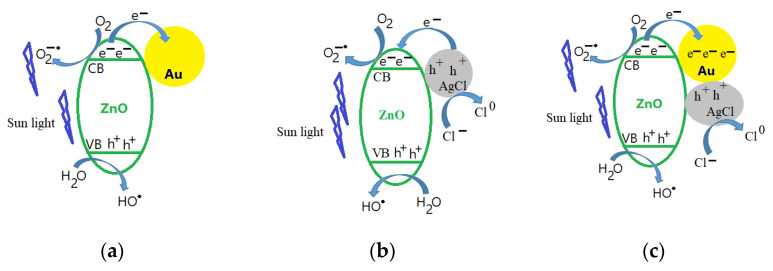
Schematic representation of the RhB degradation in the presence of the composite (**a**) AuZnO, (**b**) AgClZnO, and (**c**) AuAgClZnO.

The specialized literature [58] attributes the increase in the photocatalytic activity of the AuZnO composite, compared to ZnO, to gold, which is playing the role of an electron trap in the composite. When the ZnO surface is illuminated with visible light (see Figure 9a), the electrons from the valence band (VB) move to the conduction band (CB), while in the valence band remain the holes. The Au nanoparticles act as an electron trap from the conduction band of ZnO and, in this way, the recombination process of the electron and hole is inhibited and then enhances the photocatalytic efficiency of the AuZnO composite. In the case of the AgClZnO composite, charge carriers are generated under visible-light irradiation in AgClNPs. As the energy level of the CB of silver chloride nanoparticle is higher than that of zinc oxide particles (see Figure 9b), the photogenerated electrons are transferred from the CB of AgClNPs to that of ZnO particles [56]. Thus, the efficient separation of electron–hole pairs is achieved and leads to more reactive species. A plausible mechanism for the photodegradation of RhB in the presence of AuAgClZnO composites is illustrated in Figure 9c. Under visible-light irradiation, Au nanoparticles can absorb visible-light photons due to surface plasmons. The absorbed photons are rapidly separated into electrons and holes. Electrons are injected into the conduction band of ZnO, while holes are delivered to the surface of AgClNPs [59]. The holes from the surface of AgClNPs can lead to the oxidation of Cl^−^ ions to Cl^0^ atoms. Furthermore, the holes react with the water molecules and create the ^●^OH radical and the electrons from the conduction band react with the absorbed O_2_ and create the O_2_^−●^ radical which are the active species used in the degradation of dyes [43,58]. Superoxide radical anions, ^●^OH radicals and Cl^0^ atoms are strong oxidants for the degradation of RhB [59,60]. In the case of the three-component sample, AuAgClZnO, the obtained result can be explained based on the SEM images (Figure 6). Thus, from Figure 6, we observe that the dimensions of the AuAgClZnO structures were larger compared to those of AgClZnO. Under these conditions, the active surface of AuAgClZnO structures is smaller than in the case of AgClZnO structures and has a possible effect on the reduction in the photocatalytic activity for the tricomponent composite. Moreover, it is possible that the photocatalytic effect of the AuAgClZnO sample was lower than that of the AgClZnO sample due to the presence of Au nanoparticles.

The AuAgClZnO system presented a reaction rate constant 7.3 times higher than ZnO and 2.4 times higher than AuZnO. Similar results regarding the high photocatalytic capacity of the ternary system compared to the binary AuZnO system were obtained by Koga et al. [61], who chemically obtained nanoparticles deposited on paper, and then used them for the degradation of p-aminophenol and p-nitrophenol.

### 3.7. Evaluation of Antioxidant and Antibacterial Properties of Phytoderived Materials

The *in vitro* antioxidant activity was assessed using ABTS and chemiluminescence methods.

**Figure 10:**
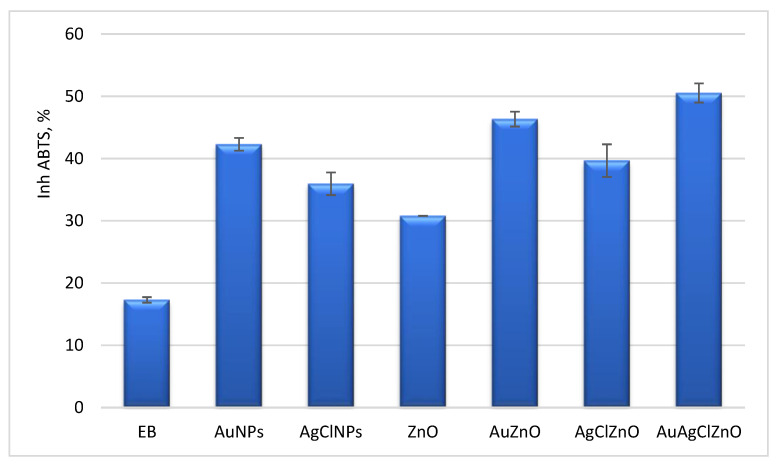
The scavenger activity of ABTS^•+^ by phytometallic particles.

Free radicals are responsible for the production of oxidative stress, and “green” nanoparticles have demonstrated that they can combat this phenomenon [62]. The capacity of inhibition of the short-life free radicals (ROS) and long-life radicals (ABTS^●+^) was comparatively evaluated for samples using two spectral methods. The samples had a capacity to capture ABTS^●+^ radicals between 30.5–50.5% higher than the burdock extract, which captured only 17% of these radicals. The best result was registered for the AuAgClZnO sample (Figure 10). The percent of ABTS^●+^ inhibition of the phyto-ZnO sample was 30.8% at 10 μg/mL, higher than that reported by S. Loganathan et al. for ZnO using *Barberis aristata* leaf (40% at 80 μg/mL). In another study, Sharmila et al. reported for green ZnO particles synthesized with *Tecoma castanifolia* leaf extract a capacity of 13% at 10 μg/mL [63]. Phyto-AuNPs showed a capacity to inhibit this radical of 42%, compared to an inhibition of 29.42% at a concentration of 100 μg/mL for gold nanoparticles synthesized with the help of *Citrullus lanatus* peels [64].

**Figure 11:**
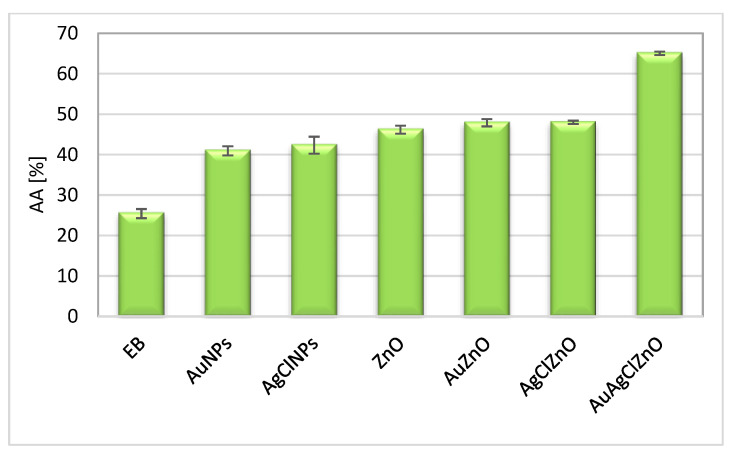
Antioxidant activity of phytometallic particles, evaluated using the chemiluminescence technique.

The chemiluminescence assay evaluates the inhibition capacity of reactive oxygen species (ROS) derived from hydrogen peroxide [35]. The antioxidant activity of the samples evaluated by chemiluminescence was between 42–65% above the activity of the native extract (AA = 25.4 ± 1.14%). In Figure 11, no significant differences were observed in the capture of ROS radicals between the dual-nanoparticle systems (AuZnO and AgClZnO) that presented values of 47%. The best result was obtained in the case of the AuAgClZnO sample, which showed an antioxidant activity of 65 ± 0.43%.

The antioxidant activity of green nanoparticles based on ZnO, silver and gold nanoparticles is explained by the presence of phytoconstituents from the burdock extract and the nanostructures with a nanosized effect that leads to the generation of many reaction centers for the capture of free radicals.

The antimicrobial activity of the burdock-generated samples was demonstrated towards three bacteria (*Escherichia coli*, *Pseudomonas aeruginosa* and *Staphylococcus aureus*), because of their high pathogenicity. Some of the bacteria in drinking water can harm the body, while others can be harmless. Some of them can be removed with the help of a water filter, while others can only disappear by treating the cause of the source of the water. The best-known bacterium that can be identified in drinking water is *Escherichia coli*, known as *E. coli*, a bacterium that can also be found in food. Each country has its own indicators, within the limits that the water meets the necessary conditions to be consumed. *E. coli* bacteria can seriously affect the body’s functioning and can cause gastroenteritis, urinary tract infections, meningitis, and septic shock [65]. Another bacterium that can be found in infected drinking water is *Pseudomonas aeruginosa* which can cause disease in animals and in humans. *Pseudomonas aeruginosa* is found in soil, water, flora, and most human environments around the world [66,67]. Rarely, another pathogenic bacterium found in water is *Staphylococcus aureus*; it produces enterotoxins that can be fatal to those who consume contaminated water or food [68,69]. Table 2 presents the antimicrobial susceptibility of the mentioned microorganisms to the tested samples.

**Figure 12:**
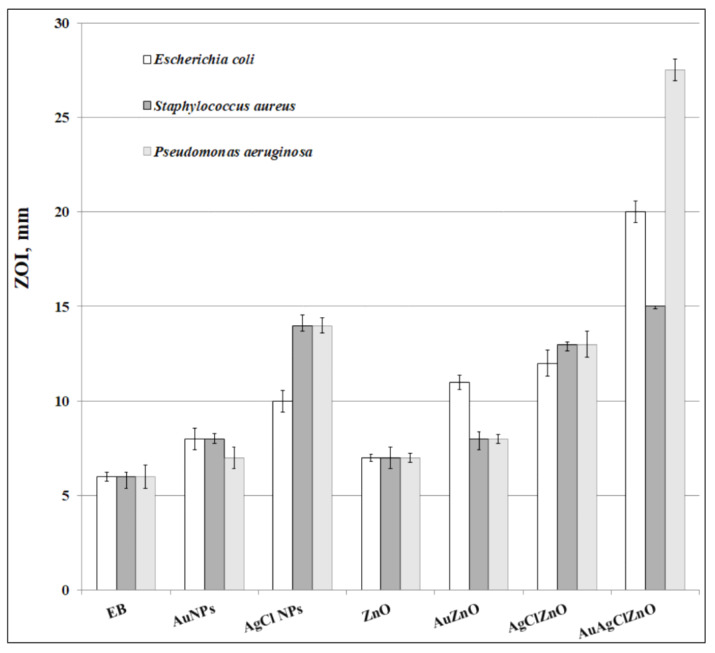
Antibacterial activity (in terms of ZOI values, mm) of burdock-derived samples.

Figure 12 displays, comparatively, the ZOI values of the prepared samples.

The antibacterial results showed that our tested samples had a better antimicrobial effect against Gram-negative bacteria because Gram-positive bacteria have a strong, thick cell wall, whereas Gram-negative bacteria have a thin cell wall, so the antibacterial agent easily penetrates and causes damages to the bacterial cell.

ZnO is a material with a broad antimicrobial spectrum with long-term antibacterial activity and minimal cytotoxicity, and it can destroy the microorganisms without the possibility of adaptation [70,71]; it is approved by the Food and Drug Administration (FDA) as a bio-safe material [72,73].

When ZnO particles come into contact with a microorganism, many mechanisms of action are possible: (1) degradation of the cell membrane via ROS (reactive oxygen species), causing lipid peroxidation and DNA/RNA damage [73]; (2) interaction between ZnO particles and the bacterial wall due to the loss of cell integrity [74]; (3) the release of Zn^2+^ ions [74].

According to Sathiyaraj et al. [75], the antibacterial activity of AuNPs was achieved in two phases: inhibition of the metabolic pathway *via* changing the membrane potential and lowering adenosine triphosphate synthase activity and, second, AuNPs rejecting the ribosome’s subunit for tRNA binding.

The antimicrobial activity of AgClNPs has been highlighted by many authors [76,77].

Our burdock-derived ZnO and AgClZnO structures were more effective against *Pseudomonas aeruginosa* (MIC_ZnO_ = 200 µg/mL; MIC_AgClZnO_ = 100 µg/mL) than the ZnO–NPs and Ag–ZnO heterostructures prepared by Hameed et al. from aqueous extract of *Silybum marianum* (MIC_ZnO-NPs_ = 250 µg/mL; MIC_Ag-ZnO_ = 150 µg/mL) [54].

Our results can be attributed to synergism between all the components of the prepared samples. Based on these results, it was concluded that burdock-derived samples could be used to combat antibiotic drug resistance.

## 4. Conclusions

This study described an original “green” bottom-up strategy to prepare multifunctional mono-, bi-, and trimetallic composites based on burdock-derived gold, silver chloride, and zinc oxide nanoparticles.

The mechanism of bio-nanomaterials’ formation was explained by correlating the UV-Vis, FTIR, and EDS spectra with information provided by microscopic images (SEM, TEM). The EDS spectra, SAED, and XRD patterns of the samples were well-correlated and showed the presence of potassium chloride in the burdock extract, and also in the AuNPs and AgClNPs. Moreover, the XRD results were well-correlated with the SAED and FTIR data, regarding the particles’ phase. The DLS results were in good agreement with the SEM and TEM analyses related to the dimensions of the obtained materials.

The optical characterization (FTIR and UV-Vis) of phytoderived materials demonstrated the key role played by proteins, flavones, polyphenols, ethers, and other phyto-compounds arising from *A. lappa* extract in the development of prepared particles. The presence of these phytomolecules gives the antioxidant and antibacterial properties and also the physical stability of our developed materials. The phytometallic and semiconducting materials presented good physical stability with zeta potential values between −18 and −35 mV. The most stable samples proved to be AgClNPs and AuAgClZnO.

The novel material AuAgClZnO showed an ability to inhibit both kinds of short-life oxygenated radicals (ROS) and long-life cationic radicals (ABTS^•+^), and an inhibition capacity of 50.5% ABTS^•+^ was determined, while the ability to capture ROS radicals was of 65%. This tricomponent system showed excellent antimicrobial properties against *Escherichia coli* (ZOI = 20 ± 0.57 mm), *Pseudomonas aeruginosa* (ZOI = 27.5 ± 0.41 mm), and *Staphylococcus aureus* (ZOI = 15 ± 0.14 mm). Additionally, the bicomponent material, AgClZnO, showed good photocatalytic properties demonstrated by the degradation of 97.02% of RhB, and antibacterial activity against the tested Gram-negative and Gram-positive bacteria. The ZnO-containing samples presented photocatalytic activity, but the most efficient system proved to be AgClZnO which can be used as an efficient catalyst in wastewater treatment for dye pollutant degradation.

The “green” developed composite particles combine the properties of all the components they are made of. Due to a synergic action of all components, the trimetallic AuAgClZnO particles presented the best bioperformance (good antioxidant and antibacterial activities) as compared to the bi- and monometallic components.

Our developed burdock-derived composites based on phytogenic gold, silver chloride, and zinc oxide particles could be used as green multifunctional platforms in various applications in the biomedical field (as antioxidant and antibacterial agents) or in environmental protection (as antimicrobial agents and catalysts for dye degradation).

## Figures and Tables

**Table 1:** The reaction rate constant k and the correlation coefficient R^2^.

Sample	k (min^−1^)	R^2^
ZnO	1.07 × 10^−3^	0.98984
AuZnO	3.27 × 10^−3^	0.99813
AgClZnO	22.97 × 10^−3^	0.99899
AuAgClZnO	7.88 × 10^−3^	0.99113

**Table 2:** Antimicrobial susceptibility of the microorganisms to burdock-derived particles.

Microorganism	Concentration of AuNPs Used (µg/mL)											
	400	200	100	50	25	12.5	6.25	3.125	1.56	0.78	0.39	0.195
*Escherichia coli* ATCC 8738	S	S	R	R	R	R	R	R	R	R	R	R
*Staphylococcus aureus* ATCC BAA 1026	S	R	R	R	R	R	R	R	R	R	R	R
*Pseudomonas aeruginosa* ATCC 15442	S	S	R	R	R	R	R	R	R	R	R	R
	Concentration of AgClNPs used (µg/mL)											
*Escherichia coli* ATCC 8738	S	S	S	R	R	R	R	R	R	R	R	R
*Staphylococcus aureus* ATCC BAA 1026	S	S	S	R	R	R	R	R	R	R	R	R
*Pseudomonas aeruginosa* ATCC 15442	S	S	S	R	R	R	R	R	R	R	R	R
	Concentration of ZnO particles used (µg/mL)											
*Escherichia coli* ATCC 8738	S	S	R	R	R	R	R	R	R	R	R	R
*Staphylococcus aureus* ATCC BAA 1026	S	R	R	R	R	R	R	R	R	R	R	R
*Pseudomonas aeruginosa* ATCC 15442	S	S	R	R	R	R	R	R	R	R	R	R
	Concentration of AuZnO used (µg/mL)											
*Escherichia coli* ATCC 8738	S	S	S	R	R	R	R	R	R	R	R	R
*Staphylococcus aureus* ATCC BAA 1026	S	S	R	R	R	R	R	R	R	R	R	R
*Pseudomonas aeruginosa* ATCC 15442	S	S	R	R	R	R	R	R	R	R	R	R
	Concentration of AgClZnO used (µg/mL)											
*Escherichia coli* ATCC 8738	S	S	S	R	R	R	R	R	R	R	R	R
*Staphylococcus aureus* ATCC BAA 1026	S	S	S	S	R	R	R	R	R	R	R	R
*Pseudomonas aeruginosa* ATCC 15442	S	S	S	R	R	R	R	R	R	R	R	R
	Concentration of AuAgClZnO used (µg/mL)											
*Escherichia coli* ATCC 8738	S	S	S	S	S	R	R	R	R	R	R	R
*Staphylococcus aureus* ATCC BAA 1026	S	S	S	S	R	R	R	R	R	R	R	R
*Pseudomonas aeruginosa* ATCC 15442	S	S	S	S	S	R	R	R	R	R	R	R

Key: R—Resistant; S—Susceptible/Sensitive.

## Data Availability

The data were included in the text.

## Supplementary Materials

The following supporting information can be downloaded at: https://www.mdpi.com/article/10.3390/ma16031153/s1, Table S1: FT-IR band assignment for vegetal extract and phytosynthesized materials. Refs. [78,79] are cited in the supplementary materials.
