# Supplementary material for: Burdock-Derived Composites Based on Biogenic Gold, Silver Chloride and Zinc Oxide Particles as Green Multifunctional Platforms for Biomedical Applications and Environmental Protection
Source: Materials (Basel). 2023 Jan 29;16(3):1153. doi: 10.3390/ma16031153 (PMC9919592; doi:10.3390/ma16031153)
Supplement: Supplementary file 1 [file materials-16-01153-s001.zip › materials-2141730-supplementary.pdf]

Supplementary material

Burdock-Derived Composites Based on Biogenic Gold, Silver Chloride and Zinc Oxide Particles as Green Multifunctional Platforms for Biomedical Applications and Environmental Protection

Irina Zgura [1,*], Nicoleta Badea [2,*], Monica Enculescu [1], Valentin-Adrian Maraloiu [1], Camelia Ungureanu [2] and Marcela-Elisabeta Barbinta-Patrascu [3,*]

1 National Institute of Materials Physics, Atomistilor 405A, 077125 Magurele, Romania
2 General Chemistry Department, Faculty of Chemical Engineering and Biotechnologies, University "Politehnica" of Bucharest, 1-7, Polizu Street, 011061 Bucharest, Romania
3 Department of Electricity, Solid-State Physics and Biophysics, Faculty of Physics, University of Bucharest, 405 Atomistilor Street, P.O. Box MG-11, 077125 Magurele, Romania
* Correspondence: irina.zgura@infim.ro (I.Z.); nicoleta.badea@upb.ro (N.B.); marcela.barbinta@unibuc.ro (M.-E.B.-P.)

Table S1. FT-IR bands assignment for vegetal extract and phyto-synthesized materials.

Sample	FT-IR Bands (cm^{-1})	Assignment	Ref.
EB	3321 (broad, strong)	Normal ''polymeric'' OH stretch; bending and stretching vibrations of hydroxyl groups intermolecularly hydrogen bonded, in alcohols, polysaccharides and phenolic compounds/ polyphenols; Stretching vibrations of the primary and secondary amines	[1]
	2933 (weak)	C–H anti-symmetric stretching vibration	[2]
	2871 (weak)	C–H symmetrical stretch vibration of alkyl chains	[2]
	1608 (strong)	Amide I, arising due to carbonyl stretch in proteins; Carboxylate groups ($–COO^-$); Stretching C=C (aromatic ring); Primary amine, NH bend	[1]
	1407 (strong)	Phenol or tertiary alcohol, OH bend	[2]
	1260 (strong)	Primary or secondary, OH in-plane bend; Aromatic ethers, aryl–O stretch	[2]

	1108/ 1082 (strong)	Secondary alcohol, C–O stretch;<br>Cyclic ethers, large rings, C–O stretch;<br>Antisymmetric stretching of –C–O group of polysaccharides and/or chlorophyll;<br>Secondary alcohol, C–O stretch;<br>C-O bending in esters; –C–O–C– ether	[1]<br>[2]
	767 (weak)	Aliphatic chloro compounds, C–Cl stretch	[2]
	613 (weak)	Alcohol, OH out-of-plane bend	[2]
AuNPs	3325 (broad, strong)	O–H stretching alcohols, polysaccharides and phenolic compounds/ polyphenols; Stretching vibrations of the primary and secondary amines	[1]
	2933 (weak)	C–H anti-symmetric stretching vibration	[2]
	2871 (weak)	C–H symmetrical stretch vibration of alkyl chains	[2]
	1617 (strong)	Amide I, arising due to carbonyl stretch in proteins<br>Carboxylate groups (–COO^-)<br>Stretching C=C (aromatic ring)<br>Primary amine, NH bend	[1]
	1399 (strong)	Carboxylates;<br>Phenol or tertiary alcohol, OH bend	[2]
	1115 (strong)	Alkyl-substituted ether; Primary and secondary alcohol, C–O stretch	[2]
	1020 (weak)	Primary amine, C–N stretch;<br>Aliphatic phosphates (P–O–C stretch)	[2]
	993	Aromatic phosphates (P–O–C stretch)	[2]
	828	C–O– stretch; Aromatic C–H out-of-plane bend	[2]
	767	Aliphatic chloro-compounds, C–Cl stretch	[2]
	618 (medium)	Alcohol, OH out-of-plane bend	[2]
AgClNPs	3297	O–H stretching alcohols, polysaccharides and phenolic compounds/ polyphenols; Stretching vibrations of the primary and secondary amines	[1]
	2923	C–H anti-symmetric stretching vibration	[2]
	2848 (very weak)	C–H symmetrical stretch vibration of alkyl chains	[2]
	1595 (sharp, strong)	Amide I, arising due to carbonyl stretch in proteins;<br>Carboxylate groups (–COO^-)<br>Stretching C=C (aromatic ring)<br>Primary amine, NH bend	[1]
	1355 (sharp, strong)	Carboxylate (carboxylic acid salt)<br>Primary or secondary, OH in-plane bend<br>Phenol or tertiary alcohol, OH bend<br>Amide III band in proteins	[1]
	1114 (sharp, strong)	Alkyl-substituted ether; Primary and secondary alcohol, C–O stretch	[2]
	618 (weak)	Alcohol, OH out-of-plane bend	[2]
ZnO	3578-3018 (very	Very broad intense band overlapping the frequencies of the following groups: Phe-	[1]

	broad intense band)	nols, OH stretch; Hydrogen-bonded O–H (the bending and stretching vibrations of hydroxyl groups in alcohols, polysaccharides and phenolic compounds)	[2]
	2127 (weak, broad)	Transition metal carbonyls	[2]
	1580 (strong, narrow)	Amide I, arising due to carbonyl stretch in proteins<br>Carboxylate groups (–COO^-)<br>Stretching C=C (aromatic ring)<br>Primary amine, NH bend	[1]
	1395 (strong, narrow)	Carboxylates; Phenol or tertiary alcohol, OH bend; C–H bend	[2]
	1268 (strong, narrow)	Primary or secondary, OH in-plane bend; Aromatic ethers, aryl–O stretch	[2]
	1036 (medium)	Primary amine, CN stretch; Alkyl-substituted ether;<br>Aliphatic phosphates (P–O–C stretch)	[2]
	907 (weak)	Aromatic phosphates (P–O–C stretch)	[2]
	545	Zn-O stretching vibration; hexagonal phase of ZnO	[3]<br>[4]
**AuZnO**	3554-3034 (very broad, strong)	Very broad intense band overlapping the frequencies of the following groups: Nonbonded hydroxyl groups, Phenols, OH stretch; Hydrogen-bonded O–H (the bending and stretching vibrations of hydroxyl groups in alcohols, polysaccharides and phenolic compounds)	[1]<br>[2]
	2976-2875 (weak, broad)	C–H anti-symmetric stretching vibration;<br>C–H symmetrical stretch vibration of alkyl chains	[2]
	2127 (very weak)	Transition metal carbonyls<br>C≡C stretch	[2]
	1585 (strong, sharp)	Amide I, arising due to carbonyl stretch in proteins<br>Carboxylate groups (–COO^-)<br>Stretching C=C (aromatic ring)<br>Primary amine, NH bend	[1]<br>[2]
	1381 (strong, sharp)	Carboxylates; Phenol or tertiary alcohol, OH bend; C–H bend	[2]
	1034 (strong, sharp)	Primary amine, C–N stretch;<br>Primary alcohol, C–O stretch<br>Alkyl-substituted ether, C–O stretch	[2]
	907 (medium)	Aromatic phosphates (P–O–C stretch)	[2]
	750 (shoulder)	Aliphatic chloro compounds, C–Cl stretch	[2]
	708 (weak)	Aliphatic chloro compounds, C—Cl stretch<br>Alcohol, OH out-of-plane bend	[2]
	694	Zn-O stretching vibration; hexagonal phase of ZnO	[3]<br>[4]

**AgClZnO**	3605-2923 (very broad, strong)	Very broad intense band overlapping the frequencies of the following groups: Phenols, OH stretch; Hydrogen-bonded O–H (the bending and stretching vibrations of hydroxyl groups in alcohols, polysaccharides and phenolic compounds);<br>C–H anti-symmetric stretching vibration	[1]<br>[2]
	2127 (weak, broad)	Transition metal carbonyls	[2]
	1499 (strong)	Amide II band in proteins	[5]
	1388 (strong)	Carboxylates; Phenol or tertiary alcohol, OH bend; C–H bend	[2]
	1071 (very weak)-1021	Primary amine, C–N stretch;<br>Primary alcohol, C–O stretch;<br>Alkyl-substituted ether, C–O stretch;<br>Secondary alcohol, C–O stretch;<br>Cyclic ethers, large rings, C–O stretch;<br>Antisymmetric stretching of –C–O group of polysaccharides;<br>Secondary alcohol, C–O stretch; C-O bending in esters; –C–O–C– ether	[1]<br>[2]
	734 (medium)	Aliphatic chloro compounds, C–Cl stretch	[2]
	704 (very weak)	Aliphatic chloro compounds, C–Cl stretch; Disulfides (C–S stretch)	[2]
	555 (weak)	Alcohol, OH out-of-plane bend	[2]
	519	Zn-O stretching vibration; hexagonal phase of ZnO	[3]<br>[4]
**AuAgClZnO**	3604-2923 (very broad, strong)	Very broad intense band overlapping the frequencies of the following groups: Nonbonded hydroxyl groups, Phenols, OH stretch; Hydrogen-bonded O–H (the bending and stretching vibrations of hydroxyl groups in alcohols, polysaccharides and phenolic compounds); C–H anti-symmetric stretching vibration	[1]<br>[2]
	2132 (weak, broad)	Transition metal carbonyls	[2]
	1790 (very weak)	Amide I, arising due to carbonyl stretch in proteins<br>Carboxylate groups (–COO–)	[2]<br>[6]
	1595, broad weak	Amide I, arising due to carbonyl stretch in proteins<br>Carboxylate groups ($-COO^-$)<br>Stretching C=C (aromatic ring)<br>Primary amine, NH bend	[1]<br>[2]
	1379, (broad, medium)	Carboxylates; Phenol or tertiary alcohol, OH bend; C–H bend	[2]

	1075-1025 (weak bands)	Primary amine, C–N stretch;<br>Primary alcohol, C–O stretch;<br>Alkyl-substituted ether, C–O stretch;<br>Secondary alcohol, C–O stretch;<br>Cyclic ethers, large rings, C–O stretch;<br>Antisymmetric stretching of –C–O group of polysaccharides;<br>Secondary alcohol, C–O stretch;<br>C-O bending in esters; –C–O–C– ether	[1]<br>[2]
	798; 735; 697 (very weak)	Aliphatic chloro compounds, C–Cl stretch	[2]
	559 (very weak)	Zn-O stretching vibration; hexagonal phase of ZnO	[3]<br>[4]

References

1. Barbinta-Patrascu, M.E.; Chilom, C.; Nichita, C.; Zgura, I.; Iftimie, S.; Antohe, S. Biophysical insights on Jack bean urease in the presence of silver chloride phytonanoparticles generated from Mentha piperita L. leaves, *Rom.Rep.Phys*., **2022**, 74, 605.
2. Coates, J. Interpretation of Infrared Spectra, a Practical Approach. In Encyclopedia of Analytical Chemistry; Meyers, R.A., Eds.; John Wiley & Sons Ltd: Chichester, UK, 2000, pp. 1-23.
3. Jankovic, S.; Milisavic, D.; Okolic, T.; Jelic, D. Synthesis of ZnO-Ag nanoparticles by sol-gel method. *Contemp. Mater.* **2019**, *10*, 22–27.
4. Rahman, F.; Majed Patwary, M.A.; Bakar Siddique, M.A.; Bashar, M.S.; Haque, M.A.; Akter, B.; Rashid, R.; Haque, M.A.; Royhan Uddin, A.K.M. Green synthesis of zinc oxide nanoparticles using Cocos nucifera leaf extract: characterization, antimicrobial, antioxidant and photocatalytic activity. *R. Soc. Open Sci.* **2022**, 9, 220858.
5. Ji, Y.; Yang, X.; Ji, Z.; Zhu, L.; Ma, N.; Chen, D.; Jia, X.; Tang, J.; Cao, Y. DFT-Calculated IR Spectrum Amide I, II, and III Band Contributions of N-Methylacetamide Fine Components. *ACS Omega*. **2020**, 5, 8572-8578.
6. Barbinta-Patrascu, M. E.; Nichita, C.; Badea, N.; Ungureanu, C.; Bacalum, M.; Zgura, I.; Iosif, L.; Antohe, S. Biophysical aspects of bio-nanosilver generated from Urtica dioica Leaves and Vitis vinifera fruits' extracts, *Rom. Rep. Phys.* **2021**, 73, 601.
